# Evaluation of Antibodies for Vascular Smooth Muscle Cell Characterization

**Published:** 2024-05-26

**Authors:** Edwin M. Shen, Lisa V Salmeron, Gabriela Sanchez, Kara E. McCloskey

**Affiliations:** 1 Graduate Program in Biological Engineering and Small-scale Technologies, University of California, Merced.; 2 School of Engineering, University of California, Merced.

**Keywords:** Flow cytometry, Antibodies, Cell characterization, Mural cells, Vascular smooth muscle cell

## Abstract

Flow cytometry, paired with fluorescent antibodies, is a popular method for characterizing cell phenotypes. Our laboratory is interested in deriving and characterizing vascular smooth muscle cells from embryonic and induced pluripotent stem cells, one of the few stem cell differentiation methods that remain underdeveloped. In our studies, we found that most commercially available antibodies advertised for smooth muscle cell identification using flow-activated cell scanning (FACS) were, in fact, not able to distinguish between positive and negative controls. Attempts to resolve the issues included exploring a range of incubation times, blocking reagents, staining kits, and titrating dilutions against both positive and negative control cells. In the end, we found that only the smooth muscle myosin heavy chain (SMMHC) antibody at a narrow titrating dilution range could distinctly bind to its intended epitope. Moreover, without more adequate and specific antibodies for labelling smooth muscle cells, we were not able to continue with our studies on smooth muscle cell fate.

## Introduction

Flow cytometry is a popular and powerful tool for the characterization of cells. Specifically, flow cytometry enables high-throughput, single cell quantification of size, granularity, and multiple fluorescent reporters [1,2]. Paired with antibodies that mark specific epitopes with reporters, flow cytometry allows researchers to identify and sort phenotypes with distinct marker profiles within a larger cell population. As such, flow cytometry has been used in the characterization and isolation of many heterogeneous cell [3–5] and stem cell-derived populations [6–8].

Critical to the accurate measurement of epitope presence is the optimization of antibody staining parameters. Because cells exhibit autofluorescence and antibodies exhibit non-specific binding, the signal-to-noise ratio must be optimized for each experiment. Typically, antibody concentrations need to be titrated to maximize signal for positive control samples relative to the background signal [9]. Negative control samples, and sometimes isotype controls, are used to exclude the possibility of non-specificity [10]. Nonspecific binding can be further mitigated with the introduction of blocking reagents that compete for nonspecific binding sites [11,12]. For example, fragment crystallizable (Fc) receptors found on many cells bind antibodies via their constant Fc domain rather than the antigen binding fragment (Fab), leading to false positives and meaningless data. In order to prevent this type of binding, Fc blocking immunoglobulins from the matching species can ensure that only specific Fab is the only binding domain observed [12]. Serum is another regularly used blocking reagent, but potentially contains lower levels of immunoglobulin compared with Fc blocking immunoglobulin.

Despite these best practices, commercial antibodies can still be reported to be nonspecific [13–16]. Berglund et al. validated over 5,000 commercially available antibodies with immunohistochemistry and Western blot experiments and found that approximately half were defective [17]. Other studies have detailed the frustrations and wasted resources of researchers who have stumbled upon faulty reagents [18,19]. This has led to the need that antibodies be rigorously validated in the context of each experiment [20,21].

Our laboratory is most interested in vascular and cardiovascular stem cell differentiation, including studies on vascular smooth muscle fate from vascular progenitor cells. The most common markers for identifying VSMC are intracellular contractile markers with no cell surface markers available for vascular smooth muscle cell identification for live cell sorting. During embryonic development, nascent VSMC first express the early marker alpha smooth muscle actin (αSMA) [22] and later, the intermediate marker calponin-h1 [23,24]. However, αSMA is also expressed in a number of other cells including myofibroblasts [25] and both αSMA and calponin-h1 are expressed in early cardiomyocytes and skeletal muscles [26,27]. Despite its ubiquitous expression, most researchers still include the expression of αSMA in their SMC characterizations, so it is included here as well. Specific, mature markers include the smooth muscle myosin heavy chain (SMMHC) [28] and smoothelin-B (SMTNB) [29], but of these markers, only smoothelin-B distinguishes vascular smooth muscle from visceral smooth muscle.

Here, we report our findings on a variety of antibodies advertised for characterization of vascular smooth muscle cells (VSMC). We perform flow cytometry tests on each antibody using primary human aortic smooth muscle cells (HAoSMC) as the positive control and non-VSMC types thought to be negative for VMSC marker expression as negative controls. We used two different commercially available intracellular staining kits and tested the effects of blocking and stain time on signal quality. Each antibody was titrated with the positive and two negative controls. Our studies indicate that out of the seven antibodies tested, only one antibody was could confidently distinguish between positive and negative controls, and only at a specific concentration.

## Materials and Methods

### Cell Sources

HAoSMC (Lonza) were used as positive controls for the anti-SMC marker antibodies. Negative controls consisted of human umbilical vein endothelial cells (HUVEC, Lonza), human induced pluripotent stem cells (hiPSC, WiCell), as well as Jiyoye, Jukrat, K562, and U937 cells (donated from Escape Therapeutics). HAoSMCs were expanded in SmGM-2 BulletKit (Lonza), HUVECs were expanded in EGM-2 BulletKit (Lonza), and hiPSC were expanded on hESC-certified Matrigel (Corning) in mTeSR1 (STEMCELL Technologies).

### Staining Protocol

Cells were fixed and stained immediately after thawing from cryopreservation. Fixation and staining methods followed the manufacturer protocol of each staining kit (eBioscience Foxp3 Staining Kit and Biolegend Transcription Factor Buffer Set). Unless otherwise stated that cells were analyzed with Viability Fixative e780, cells were fixed for 10–15 minutes, blocked with either 0.5% Human Fc Block (Biolegend) or 2% FBS (Corning) for 30–60 minutes, stained overnight at 4°C, and if applicable, stained with secondary antibodies for 30 minutes at 4°C. All primary samples are matched to a corresponding isotype and unstained sample. All isotype and secondary antibodies are used at the same IgG concentration. All samples were processed at 100,000 cells per 100 μL. Table 1 details the catalog numbers of each primary antibody, matching isotype controls, and secondary antibodies. Viability Fixative e780 (eBioscience) was used at 1:1000 concentration.

### Flow Cytometry and Data Analysis

Cells were analyzed on a LSR II flow cytometer (BD Bioscience) and data was processed on FlowJo (FlowJo). Datapoints with very low forward scatter (FSC) and side scatter (SSC) were gated out to exclude debris and very high forward scatter (FSC) were gated out to exclude doublets. Signals from the stain were gated at starting above the last 5% of the isotype signal. Samples with highly irregular data (i.e. fluorescent intensities lower than unstained controls) were omitted.

## Results and Discussion

Our original intent was to debug the issues related to staining our hiPSC-derived VSMC. Therefore, we tested antibodies for specificity by validating antibodies against HAoSMCs, which should be positive for all VSMC markers. We also tested hiPSCs, HUVECs, and several blood cell lineages, which should be negative for VSMC markers [28,30–33].

**Figure 1:**
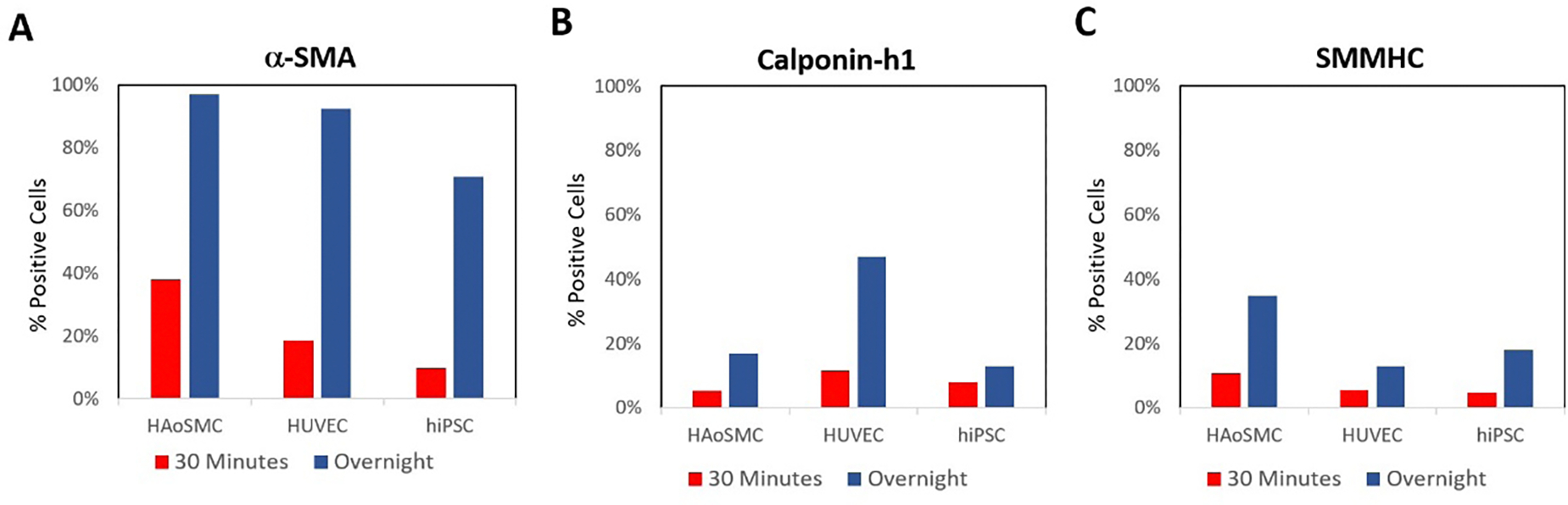
The effect of incubation time on VSMC antibody staining. Here, we stained HAoSMCs as our positive control and HUVECs and hiPSCs as negative controls. A) The effects of incubation time on αSMA (eBioscience) antibody staining. B) The effects of incubation time on calponin-h1 (Invitrogen) antibody staining. C) The effects of incubation time on SMMHC (eBioscience) antibody staining. All staining was conducted at 1:100 concentrations using the Foxp3 staining kit with no blocking.

We began by testing the effects of primary antibody incubation time (Figure 1A–C) on three antibodies, anti-αSMA (eBioscience), anti-caplonin-h1 (Invitrogen), and anti-SMMHC (eBioscience). We compared the 30 minute incubation time recommended by the Foxp3 staining kit manufacturer for intracellular staining against an overnight incubation. While the overnight incubation increased an otherwise low detection signal from all three antibodies used to stain the HAoSMC positive controls, it also undesireably increased the signals from our two negative controls. We decided to use the overnight incubation in proceeding experiments since it was able to raise the overall expression levels of the positive cell type.

**Figure 2:**
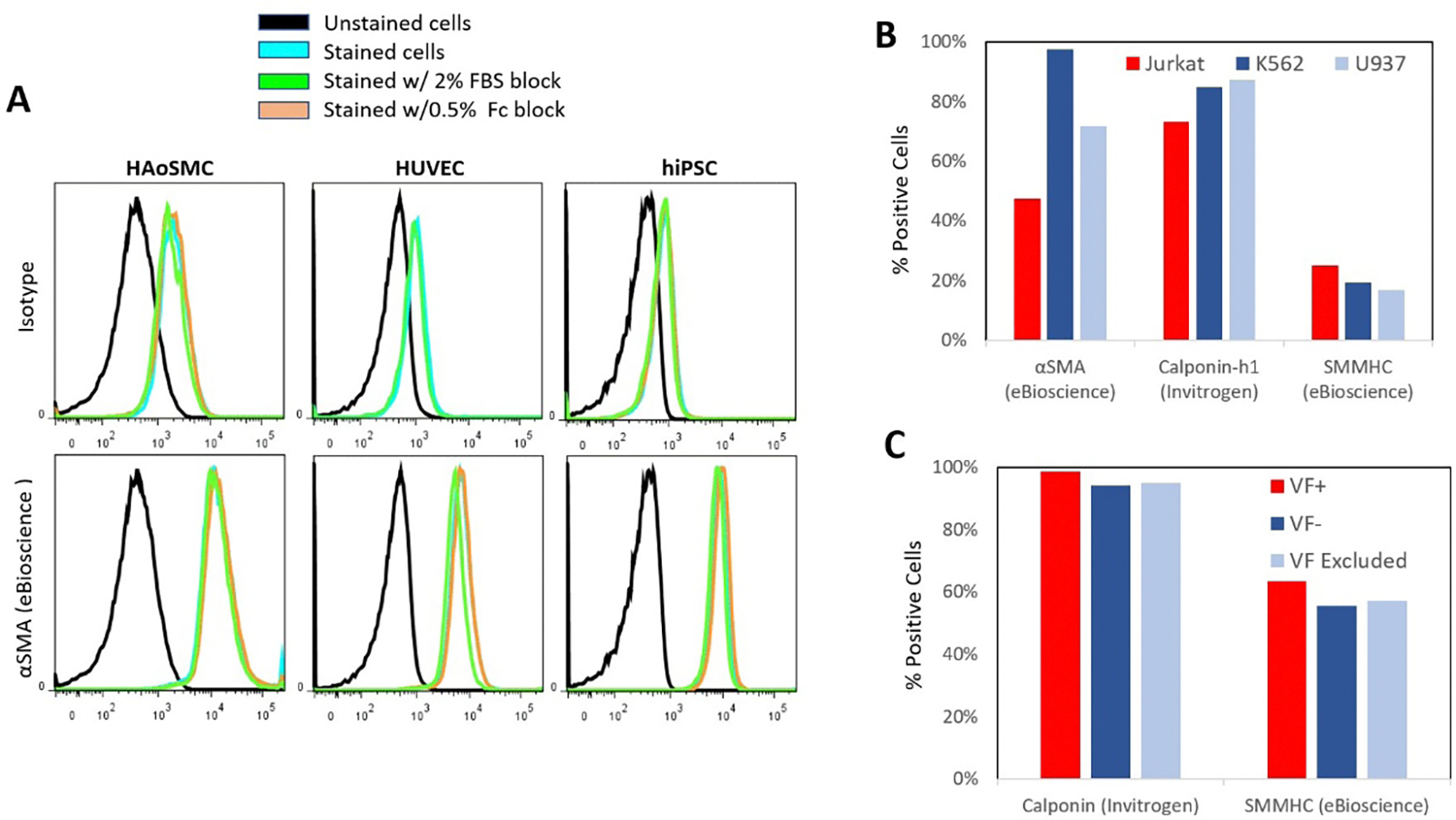
The effect of blocking reagents on VSMC antibody staining. A) The effect of blocking reagents on α-SMA antibody (eBioscience) staining positive HAoSMCs and HUVECs and hiPSCs two negative cell controls. Cells were stained with the primary antibody or isotype control and blocked for 30 minutes with either 2% FBS or 0.5% human FC receptor block. Staining was conducted at 1:100 concentrations using the Foxp3 staining kit. B) Three different VSMC marker antibodies αSMA (eBioscience), calponinh1 (Invitrogen), and SMMHC (eBioscience) were additionally examined using Jukat T-cells, K562 bone marrow cells, and U937 macrophages as negative controls. Cells were blocked with 2% FBS before overnight incubation at 1:100 concentrations with the Foxp3 kit. C) hiPSC were stained with the Transcription Buffer Staining Kit using 1:200 Calponin (Invitrogen) and 1:100 SMMHC (eBioscience) in conjunction with Viability Fixative e780. Marker expression is analyzed on populations that were positive, negative, or without viability gating.

We then examined the effects of a few different blocking buffers on the signal quality of our poorest performing antibody, αSMA (eBioscience, Figure 2A), and its corresponding isotype control. Blocking with either 0.5% Human Fc Block (Biolegend) or 2% FBS (Corning) yielded no change in signal compared to unblocked samples.

HUVECs have been reported to transdifferentiate into myoblast or VSMC-like phenotypes under abnormal culture conditions [34–36]. And hiPSC, being pluripotent, can differentiate into any cell type in the body. Although unlikely, it is possible that the VSMC markers could at times be expressed on hiPSC and HUVEC negative controls. Therefore, we additionally tested Jurkat T-cells, K562 bone marrow cells, and U937 macrophages as three additional negative cell lines and found the same high level of nonspecific binding from αSMA (eBioscience), calponin-h1 (Invitrogen), and to a lesser extent, SMMHC (eBioscience) antibodies (Figure 2B). Protocols often mention that dead cells can cause significant background signal via autofluorescence, so we tested our marker expression in conjuncation with Viability Fixative (VF). Marker expression was compared between VF+ (dead), VF− (live), and whole populations (Figure 2C). While VF+ populations exhibited marginally higher expression, it was not at a level that could account for the high amount of nonspecificty shown in this study.

**Figure 3:**
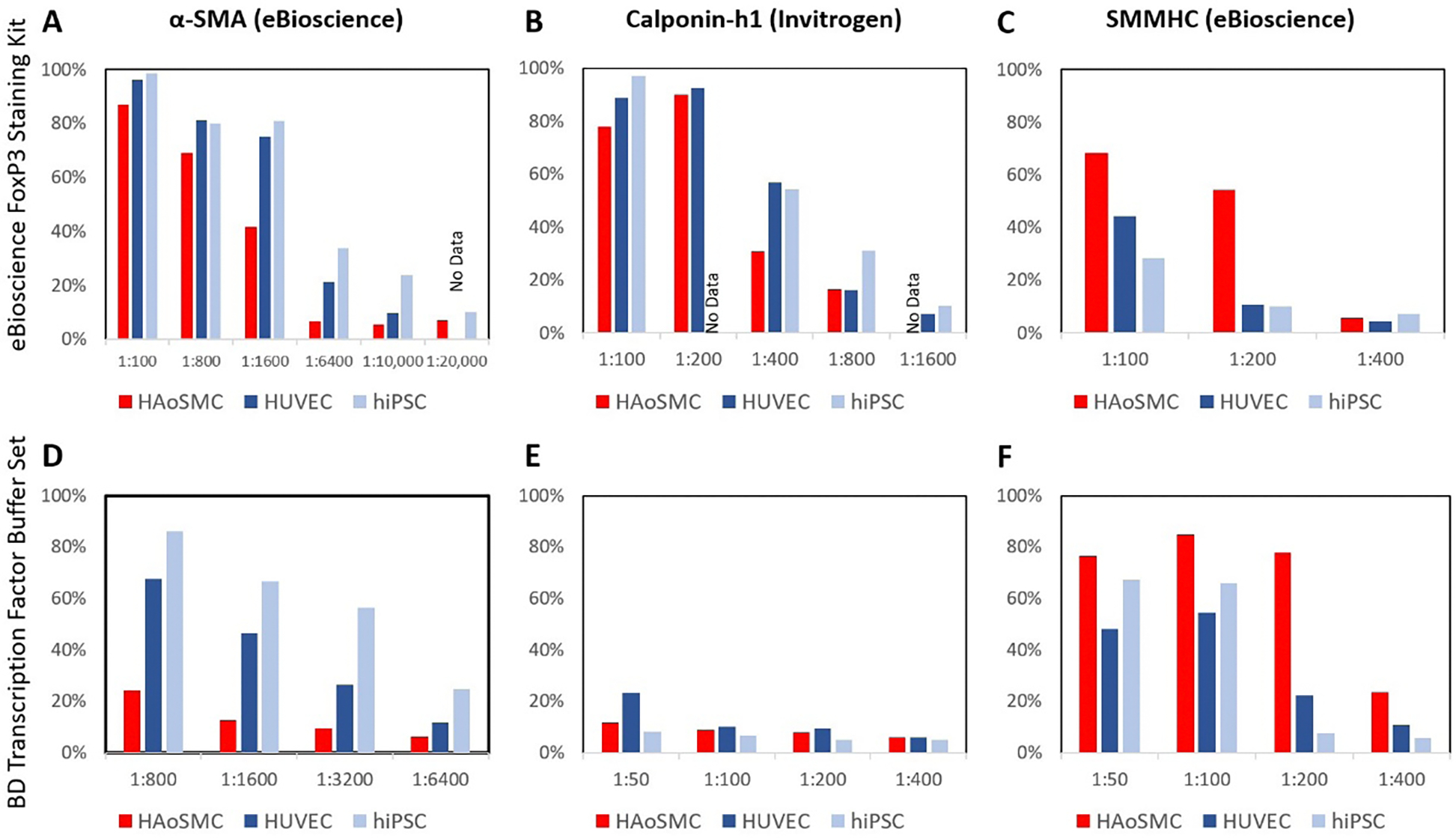
The effect of staining kits and titration on VSMC antibody staining. Antibodies α-SMA (eBioscience), calponin-h1 (Invitrogen), and SMMHC (eBioscience), respectively, were titrated against positive (HAoSMC) and negative (HUVEC and hiPSC) controls with A-C) Foxp3 staining kit with 2% FBS as the blocking buffer or D-F) Transcription Factor Set with 0.5% Human Fc Block.

Next, we performed titrations on each of the three antibodies against both positive and negative controls with two different staining kits: eBioscience Foxp3 Staining Kit and the BD Transcription Factor Buffer Set (Figure 3). Primary and isotype matched antibodies exhibited typical titration dose responses, where higher concentrations of staining material led to increased numbers of cells expressing a fluorescence signal. Among all three antibodies and both kits, only the SMMHC antibody (eBioscience) was able to distinguish correctly between positive and negative controls, however the window in which the antibody exhibited the correct staining was very small, only at the 1:200 concentration (Figure 2C&F). However, the SMMHC antibody exhibited this same consistency across both labeling kits, but reported large differences in the % positive cells with 80% reported from the BD Transcription Factor Buffer Set and only 55% positive from the eBioscience Foxp3 Staining Kit. Moreover, both the calponin-h1 and αSMA antibodies report more positive expressing cells in the negative controls compared to the positive control cells across almost all titrating dilutions.

**Figure 4:**
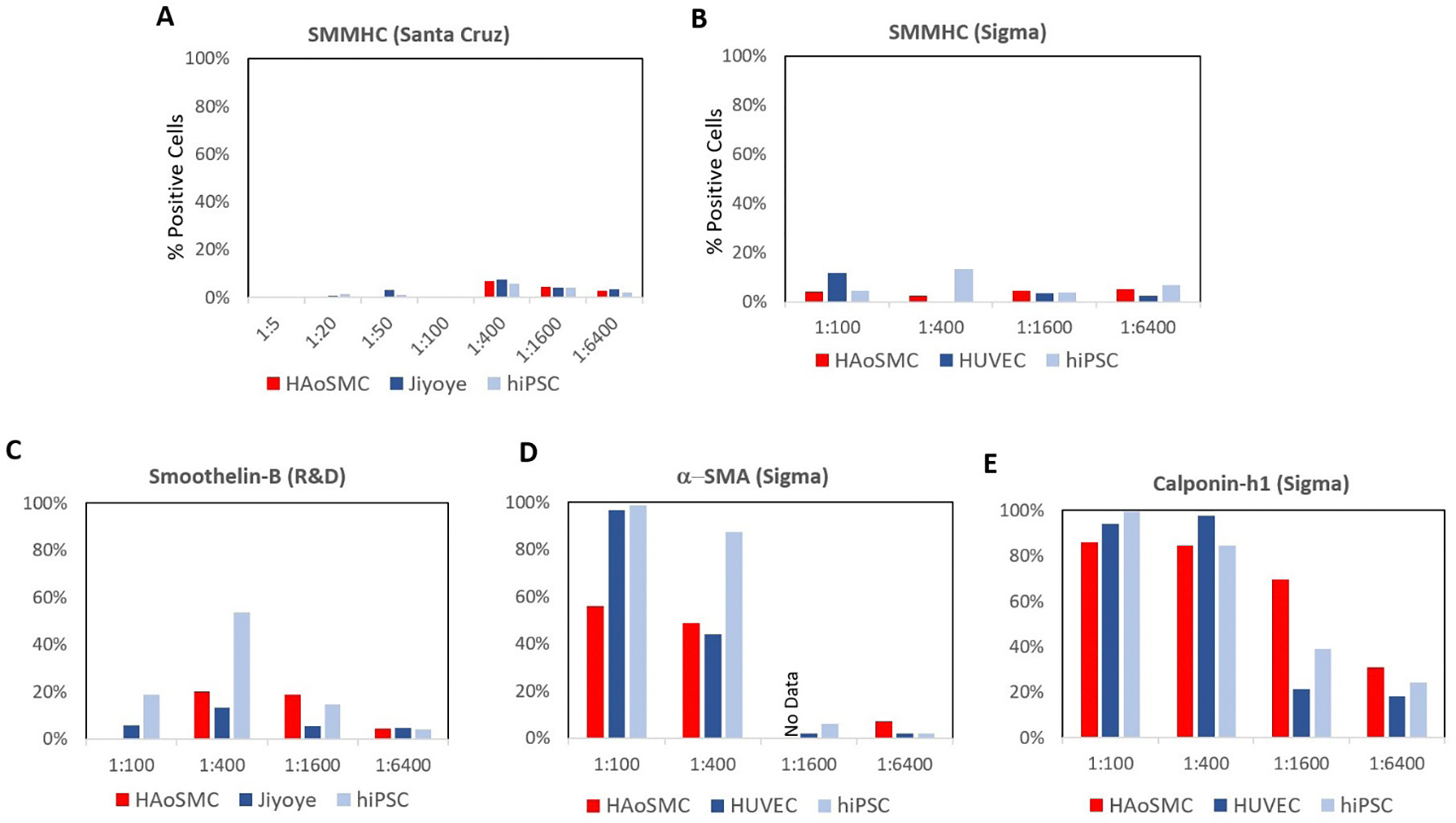
The effect of titration on more VSMC antibody staining. Dilution for SMMHC antibody from A) Santa Cruz, B) Sigma, C) smoothelin-B (R&D), D) α-SMA (Sigma), and E) calponin-h1 (Sigma). All antibodies were titrated against positive and negative controls with Foxp3 staining kit. SMMHC (Santa Cruz) and smoothelin-B (R&D) were blocked with 2% FBS and 2% Human Fc for 30 minutes. SMMHC (Sigma), α-SMA (Sigma), and calponin-h1 (Sigma) were not blocked.

We proceeded with the use of the Foxp3 kit to titrate 5 additional SMC antibodies (Figure 4). The two additional SMMHC antibodies (Santa Cruz Figure 4A and Sigma Figure 4B) examined expressed low signal on positive controls compared to the negative control cells. We also titrated anti-SMMHC (Santa Cruz) at a higher concentration (1:5–1:50), but that only exacerbated the issue (Figure 4A). Additionally, neither smoothelin-B (R&D Figure 4C) nor αSMA (Sigma Figure 4D) could distinguish between positive and negative controls at any titrating dilution. Only calponin-h1 (Sigma Figure 4E) at 1:400 was able to distinguish between positive and negative cell controls. However, the negative controls’ expression was still too high, reporting 21% and 39% for HUVECs and hiPSCs, respecitvely. Although Cheung et al. were able to distinguish between their stem cell-derived VSMCs from PSC and HUVEC negative controls, using 1:500 αSMA (Sigma), 1:30,000 calponon-h1 (Sigma), and 1:500 SMMHC (Sigma) antibodies using the BD Cytofix kit [32], their results were published in 2012, and likely used a different batch of antibodies [33–36].

## Conclusions

In summary, the SMMHC antibody (eBioscience) exhibited the best signal, though only at the 1:200 concentration (Figure 3C&F). Calponin-h1 (Sigma) also exhibited a positive signal on the positive control cells, but the difference in signal between positive and negative controls remained indistinct (Figure 4E). Moreover, many blocking buffers do not necessarily mitigate nonspecific binding and cannot be relied upon without further investigation, and incubation time and different staining protocols and kits can have a noticeable impact on the signal output of antibodies and should be considered during antibody evaluation and optimization. Based on these results, we recommend that researchers not only titrate antibodies for maximum signal, but also titrate using both positive and negative cell controls. Unfortunately for our work on deriving SMCs from hiPSCs, the one antibody that we find acceptable to use is not enough to proceed with SMC characterization.

## Figures and Tables

**Table 1:** Detailed list of antibodies with vendors and catalogue numbers, matching isotype controls, and secondary antibodies used in this study.

Intended Epitope	Primary	Advertised for FACS	Isotype Control	Secondary
**αSMA**	Sigma F3777	Yes	Biolegend 400210	
**Calponin-h1**	Sigma C2687	Yes	R&D MAB002	Abeam ab6816
**SMMHC**	Sigma M7786	Yes	R&D MAB002	Abeam ab6816
**SMMHC**	Santa Cruz sc-6956	Yes	Santa Cruz sc-2866	
**Smoothelin-B**	R&D MAB8278	Yes	R&D MAB002	Abeam ab6816
**Calponin-h1**	Invitrogen MA5–11620	No	Invitrogen MG128	Invitrogen P30013 (Conjugation Kit)
**αSMA**	eBioscience 50–9760–82	Yes	eBioscience 50–4724–80	
**SMMHC**	eBioscience 53–6400–82	No	eBioscience 50–4717–80	

## Data Availability

http://flowrepository.org/id/RvFrlK8RMK4BGeNEwN4IAGjd-JrBP7wckqz2NAuNS0cIWSN4LqMaFZqrt7fvrEyIr.
